# Valproate-Associated Hyperammonemic Encephalopathy Despite Nontoxic Serum Levels and Preserved Liver Function: A Case Report

**DOI:** 10.7759/cureus.114837

**Published:** 2026-08-20

**Authors:** Iara Ferreira, Fátima Pais, Ermelinda Gonçalves

**Affiliations:** 1 Internal Medicine, Unidade Local de Saúde de Entre Douro e Vouga, Santa Maria da Feira, PRT

**Keywords:** altered mental status, antiseizure polytherapy, drug-resistant epilepsy, encephalopathy, hyperammonemia, valproate

## Abstract

Valproate-associated hyperammonemic encephalopathy (VHE) is a potentially serious adverse effect that may occur despite nontoxic serum valproate concentrations and preserved liver function. We report a 49-year-old man with intellectual disability and long-standing drug-resistant epilepsy treated with valproate, phenytoin, phenobarbital, cenobamate, levetiracetam, and several psychotropic medications. He was admitted with severe and persistent impairment of consciousness. Initial kidney and liver function were preserved, and brain computed tomography showed no acute abnormalities. Electroencephalography demonstrated diffuse and bilateral frontotemporal slowing without electrographic seizure activity. Serum ammonia was 88.1 µmol/L and subsequently increased to 126.5 µmol/L, while valproate concentrations remained therapeutic or subtherapeutic. Phenytoin was initially supratherapeutic, but correction of its concentration did not produce meaningful neurological improvement. An extended metabolic, nutritional, autoimmune, infectious, toxicologic, and hepatic evaluation revealed no alternative cause. VHE was suspected, and valproate was gradually withdrawn without levocarnitine. This was followed by sustained neurological recovery and a decrease in serum ammonia to 43.1 µmol/L. This case emphasizes the importance of measuring serum ammonia in patients receiving valproate who develop otherwise unexplained altered mental status, particularly in the setting of intellectual disability and extensive antiseizure polytherapy.

## Introduction

Valproate is a broad-spectrum antiseizure medication widely used in epilepsy. Hyperammonemia is a recognized adverse effect and may occasionally progress to valproate-associated hyperammonemic encephalopathy (VHE), a potentially serious syndrome characterized by lethargy, cognitive slowing, vomiting, focal neurological deficits, worsening seizure control, and varying degrees of impaired consciousness [1,2]. VHE may occur without acute hepatic failure and does not require a supratherapeutic serum valproate concentration [1,2].

Routine therapeutic drug monitoring generally reports total serum valproate concentration, whereas only the unbound fraction is pharmacologically active. Because valproate is highly protein-bound, total and free concentrations may become discordant in settings such as hypoalbuminemia; therefore, an apparently therapeutic total concentration does not necessarily exclude clinically relevant free-drug exposure [3]. This distinction is particularly important when total serum concentrations are used to support causal assessment.

Reported risk factors for valproate-associated hyperammonemia include intellectual disability, carnitine depletion, urea-cycle disorders, high valproate exposure, drug interactions, and antiseizure polytherapy [2]. In adults with epilepsy receiving valproate, higher valproate dose and concomitant liver enzyme-inducing antiseizure medications or antipsychotic treatment have been associated with a higher risk of hyperammonemia [4]. These clinical data demonstrate association rather than a proven causal metabolic mechanism. Because the presentation is nonspecific, VHE may be mistaken for a postictal state, nonconvulsive status epilepticus, infection, respiratory or metabolic disturbance, toxicity from concomitant medication, or medication-related sedation [2,5]. Prompt recognition is important because valproate reduction or withdrawal is the principal therapeutic intervention and is frequently followed by neurological and biochemical improvement [2,5].

We report a diagnostically complex case of VHE in a patient with intellectual disability and long-standing drug-resistant epilepsy receiving extensive antiseizure and psychotropic polytherapy, in whom measured total serum valproate concentrations were therapeutic or subtherapeutic.

## Case presentation

A 49-year-old man with intellectual disability and long-standing drug-resistant epilepsy was admitted with severe and persistent impairment of consciousness. He had been receiving valproate 2,000 mg/day for several years, with no recent dose adjustments before admission. His remaining antiseizure regimen included phenytoin 200 mg/day, levetiracetam 1,500 mg/day, phenobarbital 45 mg/day, cenobamate 200 mg/day, and clobazam 15 mg/day. Concomitant psychotropic therapy included paroxetine 40 mg/day, olanzapine 10 mg/day, quetiapine 50 mg/day, and depot haloperidol 150 mg every three weeks. On admission, he was markedly somnolent, with inconsistent eye opening, absent visual engagement, and no meaningful verbal response.

Initial laboratory testing showed no significant systemic inflammatory response, with preserved renal and hepatic synthetic function (Table 1).

**Table 1:** Laboratory findings at presentation Initial hematologic, renal, electrolyte, hepatic, endocrine, and inflammatory laboratory findings obtained during the evaluation of severe impairment of consciousness. Reference ranges correspond to those used by the reporting laboratory. INR, international normalized ratio; AST, aspartate aminotransferase; ALT, alanine aminotransferase; LDH, lactate dehydrogenase; TSH, thyroid-stimulating hormone; CRP, C-reactive protein

Parameter		Result	Unit	Reference range
Hemoglobin		14.5	g/dL	12.0-16.0
White blood cells		5.4	x10⁹/L	4.0-11.0
Platelets		235	x10⁹/L	150-450
International normalized ratio (INR)		1.2	-	-
Urea		31	mg/dL	15-40
Creatinine		0.6	mg/dL	0.6-1.1
Sodium		136	mmol/L	136.0-145.0
Potassium		4.2	mmol/L	3.5-5.1
Total bilirubin		0.24	mg/dL	0.20-1.20
Aspartate aminotransferase (AST) / alanine aminotransferase (ALT)		14/13	U/L	5-34 / 0-55
Lactate dehydrogenase (LDH)		167	U/L	125-220
Thyroid-stimulating hormone (TSH)		0.41	µIU/mL	0.40-4.00
Procalcitonin		<0.02	ng/mL	<0.1 ng/mL
C-reactive protein (CRP)		16.7	mg/L	0.0-5.0

**Figure 1:**
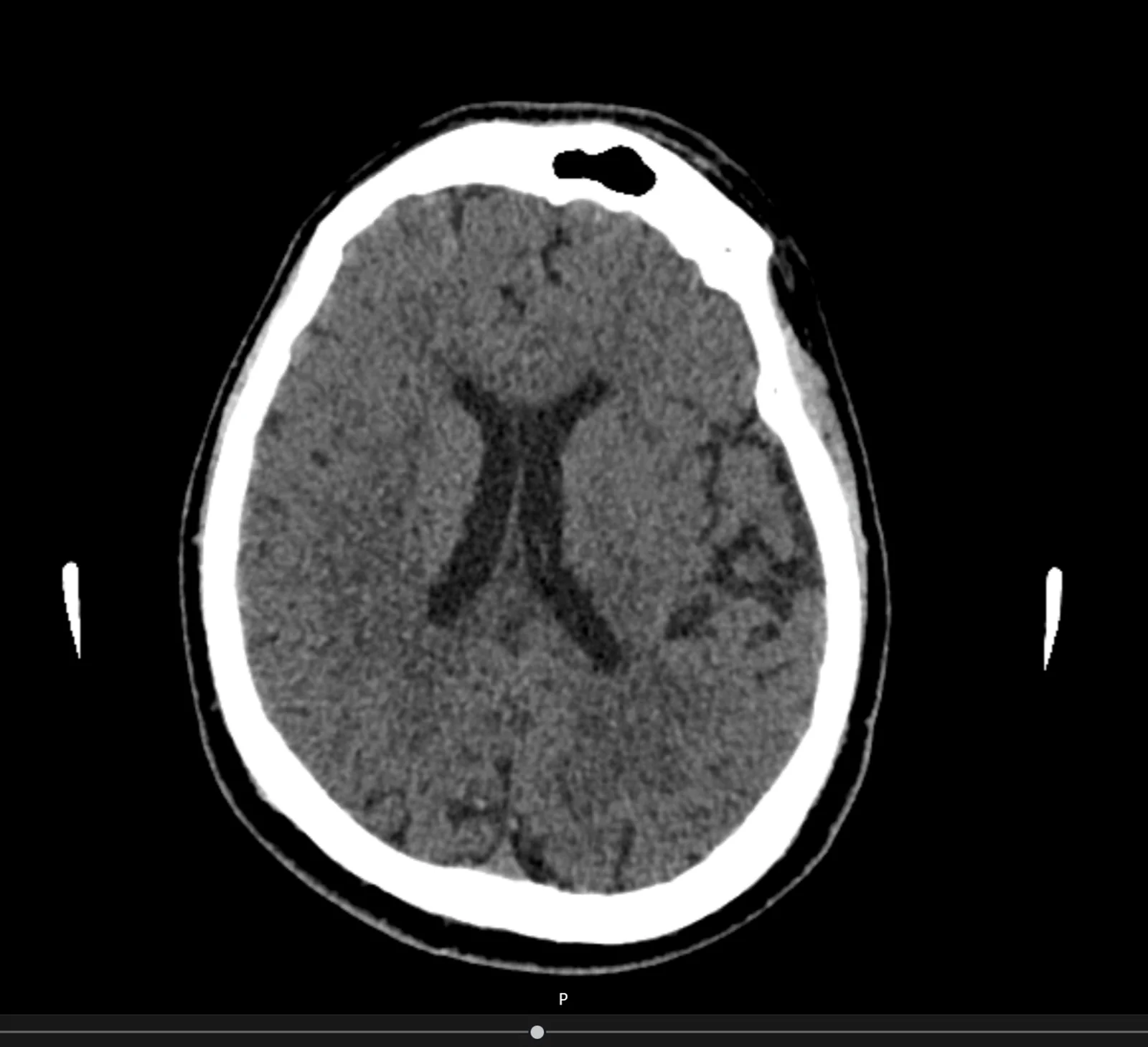
Non-contrast brain computed tomography Non-contrast axial brain computed tomography showing no acute intracranial abnormality. No evidence of acute intracranial hemorrhage, mass effect, midline shift, or hydrocephalus is identified.

Urine toxicology was positive for benzodiazepines and barbiturates, consistent with the prescribed clobazam and phenobarbital, respectively. Initial urinalysis and urine culture showed no evidence of infection. Non-contrast brain computed tomography revealed no acute intracranial abnormalities, including no evidence of hemorrhage, ischemia, or mass effect (Figure 1).

A prolonged resting electroencephalogram (EEG) showed mild diffuse background slowing, intermittent bilateral frontotemporal slowing, and isolated sharp waves, without evidence of electrographic seizure activity. The exact recording duration was not documented.

Because the patient's altered mental status remained unexplained, serum ammonia was measured and was elevated at 88.1 µmol/L. Serial measurements confirmed persistent hyperammonemia, with subsequent values of 106.1, 103.8, and 126.5 µmol/L. The measured total serum valproate concentrations were therapeutic or subtherapeutic, and phenobarbital concentrations remained within the therapeutic range. Albumin was not measured at presentation but was mildly reduced when subsequently assessed, and only total serum valproate concentrations were available; free valproate concentrations were not measured. By contrast, serum phenytoin was initially markedly supratherapeutic at 45.77 µg/mL and subsequently decreased to 35.05 and 31.56 µg/mL. Phenytoin was discontinued on hospital day three and had fallen to 19.7 µg/mL by hospital day six, but the patient remained severely encephalopathic, arguing against phenytoin toxicity as the sole explanation. Following the identification of hyperammonemia, an extended nutritional, autoimmune, infectious, and hepatic etiologic evaluation was performed. Vitamin B12 and folate levels were adequate; antinuclear antibodies, cryoglobulins, hepatitis C antibodies, and syphilis serology were negative. Immunoglobulins, alpha-1 antitrypsin, and ceruloplasmin were unremarkable. Targeted metabolic testing showed plasma alanine, arginine, and citrulline concentrations within their respective reference ranges, while urinary orotic acid was undetectable; these findings did not support a urea-cycle disorder. The available evaluation did not identify an additional metabolic, nutritional, autoimmune, or structural hepatic cause of hyperammonemia (Table 2).

**Table 2:** Laboratory and etiologic findings during the evaluation of encephalopathy and hyperammonemia Arrows indicate chronological measurements. The remaining urine toxicology screen included amphetamines, cocaine, tetrahydrocannabinol, tricyclic antidepressants, methamphetamines, morphine, MDMA, and methadone. Laboratory and etiologic findings were obtained during the evaluation of persistent altered mental status and hyperammonemia. Arrows indicate chronological measurements. Albumin was not measured at presentation. Urine benzodiazepine and barbiturate positivity was consistent with prescribed clobazam and phenobarbital, respectively. Plasma alanine, arginine, and citrulline concentrations were within their respective laboratory reference ranges, and urinary orotic acid was undetectable. IgA, immunoglobulin A; IgG, immunoglobulin G; IgM, immunoglobulin M; anti-HCV, hepatitis C antibody; S/CO, signal-to-cutoff ratio

Parameter	Result	Unit	Reference range
Serum ammonia	88.1 → 106.1 → 103.8 → 126.5 → 43.1	µmol/L	≤72
Total serum valproate	51.7 → 47.9 → 25.9 → <12.5	µg/mL	50–100
Phenytoin	45.77 → 35.05 → 31.56 → 19.72 → <1.80	µg/mL	10–20
Phenobarbital	18.8 → 18.6 → 19.4 → 16.3 → 18.4	µg/mL	15–40
Albumin	Not measured at presentation → 3.3 →3.2	g/dL	3.5-5.0
Vitamin B12	732	pg/mL	197–771
Folate	11.8	ng/mL	>4.0
Antinuclear antibodies	<0.2, negative	U/mL	Negative
Cryoglobulins	Negative	—	Negative
Immunoglobulin A (IgA)	230	mg/dL	70–400
Immunoglobulin G (IgG)	774	mg/dL	700–1,600
Immunoglobulin M (IgM)	72	mg/dL	40–230
Plasma alanine	205	µmol/L	150–550
Plasma arginine	33	µmol/L	20–150
Plasma citrulline	31	µmol/L	10–45
Alpha-1 antitrypsin	187	mg/dL	90–200
Ceruloplasmin	35	mg/dL	20–40
Hepatitis C antibody (anti-HCV)	0.01, negative	S/CO	Negative
Anti-Treponema pallidum antibody	0.02, nonreactive	S/CO	Nonreactive
Urine benzodiazepine screen	Positive	—	Negative
Urine barbiturate screen	Positive	—	Negative
Remaining urine toxicology screen	Negative	—	Negative
Urine culture	Negative	—	Negative
Urinary orotic acid	Undetectable	mmol/mol creatinine	≤2.0
Two peripheral blood cultures	Negative	—	Negative

Abdominal ultrasonography showed no structural liver disease.

On hospital day three, the patient developed fever, abundant respiratory secretions, and worsening hypoxemia, clinically considered probable aspiration pneumonia. He required supplemental oxygen and received intravenous amoxicillin/clavulanate. C-reactive protein (CRP) subsequently increased to 270.6 mg/L. The respiratory infection and gas-exchange disturbance improved with treatment, yet marked impairment of consciousness persisted.

VHE was suspected. Free and total serum carnitine concentrations could not be measured because the assay was unavailable at the hospital laboratory. Valproate was gradually tapered and completely discontinued. Neurological function subsequently improved, and serum ammonia decreased to 43.1 µmol/L. Levocarnitine was considered; however, given the neurological and biochemical recovery following valproate discontinuation, it was ultimately not administered. The overall temporal pattern supported VHE as an important contributor despite therapeutic or subtherapeutic total serum valproate concentrations and preserved hepatic synthetic function.

Aminotransferase levels increased later during hospitalization, with aspartate aminotransferase (AST) reaching 243 U/L and alanine aminotransferase (ALT) 249 U/L on hospital day 14, when serum ammonia had already normalized. Hepatic synthetic function remained preserved. The chronological relationship among neurological status, ammonia and antiseizure drug concentrations, respiratory and infectious events, laboratory findings, and therapeutic changes is summarized in Table 3.

**Table 3:** Chronological clinical, biochemical, and therapeutic evolution Drug concentrations are serum concentrations in µg/mL unless otherwise specified. Reported valproate and phenytoin concentrations are total serum concentrations; free concentrations were not available. HD, hospital day; NH₃, ammonia; VPA, valproate; PHT, phenytoin; PB, phenobarbital; LEV, levetiracetam; CNB, cenobamate; CLB, clobazam; EEG, electroencephalogram; ABG, arterial blood gas; pCO₂, partial pressure of carbon dioxide; pO₂, partial pressure of oxygen; HCO₃, bicarbonate; CRP, C-reactive protein; AST, aspartate aminotransferase; ALT, alanine aminotransferase; GGT, gamma-glutamyl transferase; INR, international normalized ratio

Hospital day / date	Neurological status	Ammonia / antiseizure drug concentrations	Respiratory / infectious course	Other investigations / liver-inflammatory data	Therapeutic changes
HD0 / July 4	Marked somnolence; inconsistent eye opening; absent visual engagement; no meaningful verbal response.	PHT 45.77 µg/mL; PB 18.8 µg/mL; ammonia not yet measured.	Admission ABG: pH 7.38, pCO₂ 48 mmHg, pO₂ 62 mmHg, HCO₃ 28.4 mmol/L; no established infection.	CRP 16.7 mg/L; creatinine 0.6 mg/dL; total bilirubin 0.24 mg/dL; INR 1.2; AST 14 U/L; ALT 13 U/L.	VPA 2,000 mg/day; PHT 200 mg/day; PB 45 mg/day; LEV 1,500 mg/day; CNB 200 mg/day; CLB 15 mg/day.
HD1 / July 5	Persistent marked impairment of consciousness.	NH₃ 88.1 µmol/L; total VPA 51.7 µg/mL; PHT 35.05 µg/mL; PB 18.6 µg/mL.	No established infection at this stage.	No hepatic synthetic failure documented.	No major VPA change documented.
HD2 / July 6	Persistent encephalopathy.	No new drug level documented.	No major respiratory event documented.	EEG: mild diffuse slowing, intermittent bilateral frontotemporal slowing, and isolated sharp waves; exact recording duration not documented.	No major medication change documented.
HD3 / July 7	Markedly impaired consciousness with respiratory deterioration.	NH₃ 106.1 µmol/L; total VPA 47.9 µg/mL; PHT 31.56 µg/mL; PB 19.4 µg/mL.	Probable aspiration pneumonia: temperature 38.8°C, abundant secretions, and respiratory deterioration; ABG pH 7.39, pCO₂ 52 mmHg, pO₂ 43 mmHg, HCO₃ 31.5 mmol/L; supplemental oxygen required.	AST 57 U/L; ALT 77 U/L; GGT 551 U/L; CRP subsequently 270.6 mg/L.	VPA reduced to 1,200 mg/day; PHT discontinued; PB reduced to 15 mg/day; intravenous amoxicillin/clavulanate started.
HD6 / July 10	Persistent severe encephalopathy despite improving respiratory status.	NH₃ 103.8 µmol/L; total VPA 26.0 µg/mL; PHT 19.7 µg/mL; PB 16.3 µg/mL.	Aspiration pneumonia improving with treatment; supplemental oxygen still required.	Repeat EEG similar; AST 68 U/L; ALT 82 U/L; GGT 501 U/L; CRP approximately 165 mg/L.	PHT remained discontinued; VPA 1,200 mg/day continued.
HD10 / July 14	Persistent encephalopathy; no meaningful verbal response.	NH₃ 126.5 µmol/L (peak).	Aspiration pneumonia clinically improving.	Albumin 3.3 g/dL; CRP 157.7 mg/L; AST 103 U/L; ALT 92 U/L; GGT 481 U/L; bilirubin 0.17 mg/dL.	Further VPA reduction ordered; levocarnitine considered.
HD11 / July 15	Persistently impaired consciousness.	No new NH₃ or VPA level documented.	Afebrile; oxygen requirement decreasing.	Albumin 3.2 g/dL; CRP 111.7 mg/L; AST 125 U/L; ALT 124 U/L; GGT 469 U/L.	VPA documented at 300 mg twice daily (600 mg/day).
HD12 / July 16	Early neurological improvement with directed gaze and intermittent command following.	No new level documented.	Respiratory episode improved.	No hepatic synthetic failure documented.	Valproate completely discontinued.
HD13 / July 17	Meaningful neurological recovery with spontaneous eye opening, recognition, verbal interaction, and simple command following.	No new level documented.	Respiratory episode resolved.	No hepatic synthetic failure documented.	Valproate remained discontinued.
HD14 / July 18	Sustained neurological improvement.	NH₃ 43.1 µmol/L; total VPA <12.5 µg/mL; PHT <1.80 µg/mL.	Initial aspiration pneumonia resolved.	CRP 117.8 mg/L; AST 243 U/L; ALT 249 U/L; GGT 476 U/L; total bilirubin 0.21 mg/dL; INR 1.3; ammonia already normalized.	Valproate remained discontinued.
HD16 / July 20	Awake after encephalopathy improvement; two clinically observed versive seizures with tonic upper-limb elevation.	No new NH₃ level documented.	No new respiratory event documented.	No new relevant liver data documented.	Topiramate 50 mg twice daily introduced.
HD17 / July 21	Vigilant and interactive; neurological improvement sustained.	Total VPA <12.5 µg/mL; PB 18.4 µg/mL.	No new respiratory event documented.	Albumin 3.5 g/dL; AST 124 U/L; ALT 238 U/L; GGT 411 U/L.	No further clinically apparent seizures after topiramate; remaining antiseizure therapy continued.
HD20–23 / July 24–27	Neurological status remained improved relative to the hyperammonemic period.	No new relevant NH₃ level documented.	Separate later febrile/inflammatory episode treated with piperacillin/tazobactam.	CRP peaked at 270.4 mg/L; procalcitonin peaked at 22.59 ng/mL.	No valproate re-exposure; antiseizure regimen continued.

**Figure 2:**
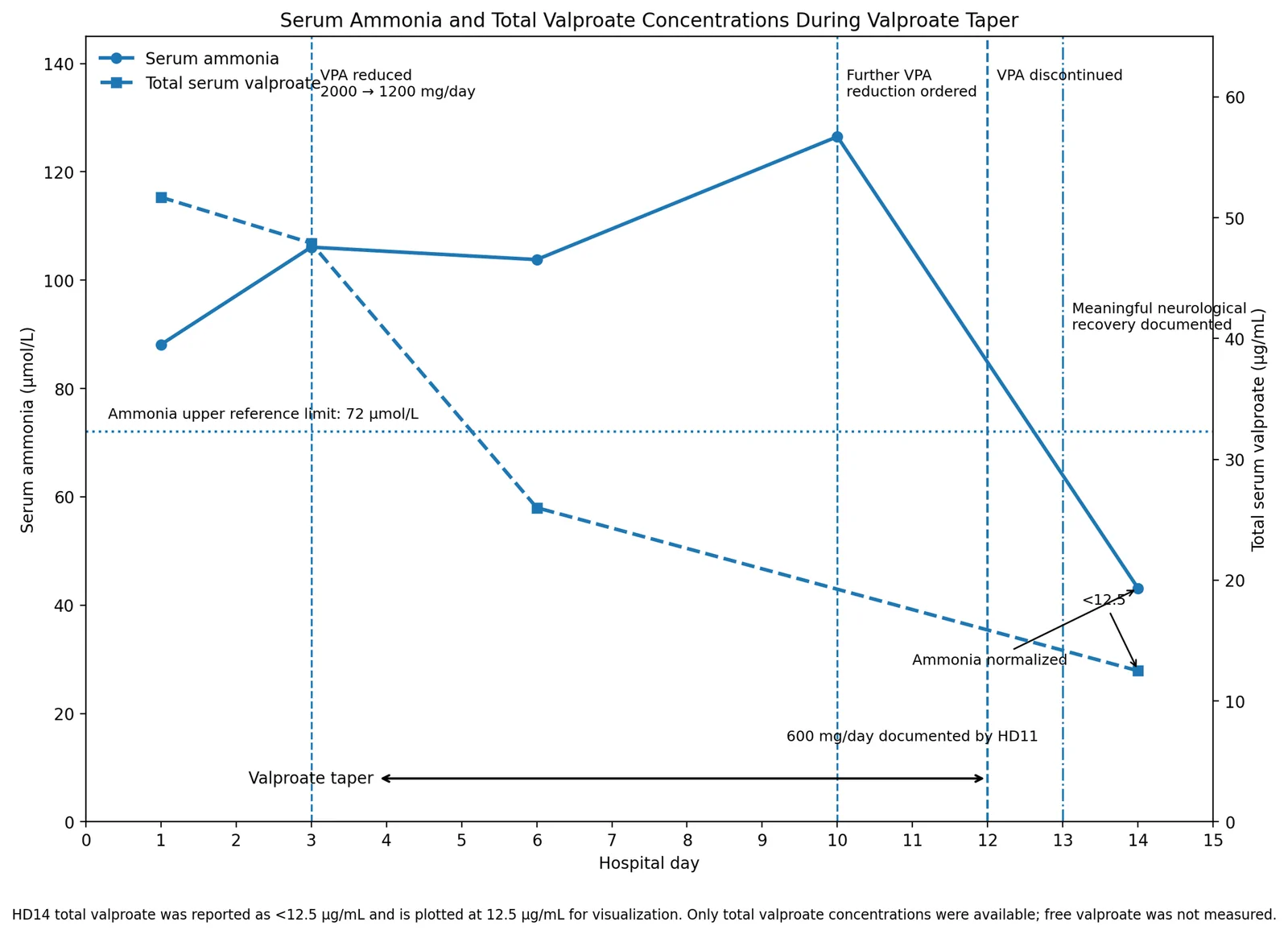
Serum ammonia and total serum valproate concentrations during valproate taper Serial serum ammonia and measured total serum valproate concentrations during hospitalization. Valproate was reduced from 2,000 to 1,200 mg/day on hospital day three, a further reduction was ordered on hospital day 10, 600 mg/day was documented by hospital day 11, and valproate was completely discontinued on hospital day 12. Meaningful neurological recovery was documented on hospital day 13, and serum ammonia normalized to 43.1 µmol/L on hospital day 14. The total valproate concentration on hospital day 14 was reported as <12.5 µg/mL and is plotted at 12.5 µg/mL for visualization. Only total valproate concentrations were measured; free valproate concentrations were not available. VPA, valproate; HD, hospital day

Serial serum ammonia and total serum valproate concentrations, together with the valproate taper, complete withdrawal, and timing of meaningful neurological recovery, are shown in Figure 2. 

Several days after valproate withdrawal, two clinically observed versive seizures with tonic elevation of an upper limb recurred. Topiramate 50 mg twice daily was added, after which no further clinically apparent seizures were documented during the subsequent hospital course.

## Discussion

VHE is an uncommon but potentially serious adverse effect of valproate [2,5]. The diagnosis is generally inferred from a compatible neurological syndrome, documented hyperammonemia, evaluation for alternative explanations, and clinical and biochemical improvement after valproate reduction or withdrawal [2,6]. Neither a supratherapeutic total serum valproate concentration nor acute hepatic failure is required [1,2]. In the present case, measured total valproate concentrations were therapeutic or subtherapeutic and hepatic synthetic function remained preserved during the principal hyperammonemic period. Aminotransferase levels subsequently increased, reaching their highest values after serum ammonia had already normalized, making acute hepatic failure an unlikely primary explanation for the hyperammonemia.

Interpretation of the measured valproate concentrations requires an important qualification. The available values were total serum concentrations. Albumin was not measured at presentation but was mildly reduced when subsequently assessed (3.3 g/dL on hospital day 10 and 3.2 g/dL on hospital day 11), and free valproate concentrations were not measured. Because hypoalbuminemia may produce discordance between total and unbound valproate, a therapeutic or subtherapeutic total concentration cannot establish that free exposure was low [3]. Free phenytoin concentrations were likewise unavailable. Nevertheless, the markedly elevated initial total phenytoin concentration, subsequent fall into the therapeutic range, and continued severe encephalopathy after that correction argue against phenytoin toxicity as the sole explanation. The systematic review by Lin et al. identifies hypoalbuminemia as a predictor of discordance between total and free valproate concentrations [3].

The pathophysiology of valproate-associated hyperammonemia is multifactorial. Valproate may interfere with ureagenesis and mitochondrial metabolism, while chronic exposure may reduce carnitine availability and impair beta-oxidation [2,7]. Carnitine depletion was therefore a plausible mechanism but was not demonstrated in this patient because free and total carnitine concentrations could not be measured locally. In addition, the association between concomitant enzyme-inducing antiseizure medications and hyperammonemia described in clinical studies is observational [4]. The present case therefore does not establish that phenytoin or phenobarbital increased production of a specific toxic valproate metabolite.

Several plausible contributors to impaired consciousness coexisted. Phenytoin toxicity was present early, aspiration pneumonia was associated with a hypoxemic-hypercapnic respiratory disturbance, and multiple sedating psychotropic and antiseizure medications were being administered. Severe underlying epilepsy and infection-associated physiological stress were additional confounders. However, neurological dysfunction persisted after phenytoin returned to the therapeutic range and as the acute respiratory deterioration improved, while ammonia remained elevated and subsequently peaked at 126.5 µmol/L. The most appropriate interpretation is therefore a multifactorial encephalopathy in which valproate-associated hyperammonemia represented an important and reversible contributor rather than the only possible cause.

Alternative causes of non-hepatic hyperammonemia were considered. Renal function remained preserved, initial urine studies and culture did not support a urease-producing urinary infection, and ultrasonography did not show structural chronic liver disease. There was no evidence of hepatic synthetic failure during the principal hyperammonemic episode, and serial EEG reports did not describe electrographic status epilepticus. Targeted metabolic testing showed plasma alanine, arginine, and citrulline concentrations within their respective laboratory reference ranges, while urinary orotic acid was undetectable. These findings did not provide a biochemical pattern supporting a urea-cycle disorder; however, late-onset urea-cycle disorders may present variably [8]. The respiratory infection and associated physiological stress remained temporally plausible contributors. These findings emphasize that VHE is a clinical diagnosis supported by the overall temporal pattern rather than by ammonia concentration alone. Plasma amino-acid profiling and urinary orotic acid are established components of the biochemical investigation of urea-cycle disorders [8].

Serum ammonia increased from 88.1 to 106.1 and 103.8 µmol/L before peaking at 126.5 µmol/L. Serum ammonia concentration does not consistently correlate with the onset or severity of VHE [2]. Moreover, a systematic review found that asymptomatic hyperammonemia is substantially more common than symptomatic hyperammonemia and may occur within the therapeutic total valproate concentration range [6]. Thus, an isolated ammonia elevation should not establish VHE; rather, the diagnosis depends on a compatible clinical syndrome, assessment of competing causes, and response to intervention. Chopra et al. report a lack of reliable correlation between serum ammonia and VHE severity [2], while Wong et al. demonstrate the higher prevalence of asymptomatic hyperammonemia and its occurrence at therapeutic valproate concentrations [6].

Valproate dose reduction or discontinuation remains the central therapeutic intervention [2,5,9]. In this patient, neurological improvement became apparent during the final taper and continued after complete withdrawal, with normalization of serum ammonia by hospital day 14. Levocarnitine was considered; however, given the neurological and biochemical recovery following valproate discontinuation, it was ultimately not administered. L-carnitine has a plausible biological rationale and may be useful in valproate-associated hyperammonemia, although the optimal dosing regimen and route of administration remain uncertain [7]. Valproate withdrawal is described as the mainstay of treatment by Chopra et al. [2]; dose reduction or discontinuation was a common successful strategy in the Vaidyanathan series [5], and Baddour et al. found valproate discontinuation to be the most effective intervention in their cohort [9].

Withdrawal of valproate also required reassessment of seizure control. Two clinically observed seizures recurred on hospital day 16, several days after complete withdrawal. Topiramate 50 mg twice daily was added, after which no further clinically apparent seizures were documented in the subsequent available record. This illustrates the therapeutic trade-off in patients with drug-resistant epilepsy: management of suspected VHE must be balanced against the risk of seizure recurrence and requires active adjustment of the remaining antiseizure regimen.

The principal evidence supporting a causal contribution from valproate was the dechallenge pattern across dose reduction and withdrawal, together with persistent hyperammonemia after correction or improvement of other major competing contributors. Definitive attribution to valproate alone is not possible. Serial Glasgow Coma Scale scores were not consistently documented and therefore were not reconstructed retrospectively; neurological evolution was instead described using contemporaneous examination findings. The exact EEG recording durations were also unavailable, free valproate and phenytoin concentrations were not measured, and carnitine status could not be assessed. Targeted biochemical testing did not support a urea-cycle disorder, although late-onset presentations are recognized [8]. Valproate rechallenge was not performed.

Routine ammonia monitoring in every asymptomatic patient receiving valproate is not supported by current evidence [6,10]. Ammonia measurement is most useful when a patient receiving valproate develops new or otherwise unexplained lethargy, cognitive deterioration, vomiting, worsening seizure control, ataxia, or impaired consciousness [2,6,10]. The present case further illustrates the value of reconstructing medication, respiratory, infectious, hepatic, and neurological timelines when several reversible causes of encephalopathy coexist. Wong et al. found insufficient evidence to support routine ammonia monitoring [6], while McMorris et al. specifically recommend measurement when altered mental status or encephalopathy develops [10].

## Conclusions

VHE should be considered when a patient receiving valproate develops otherwise unexplained altered consciousness, even when measured total serum valproate concentrations are therapeutic or subtherapeutic and hepatic synthetic function is preserved. Total valproate concentrations should be interpreted cautiously when albumin is reduced because free exposure may not be reflected accurately by the total concentration. The diagnosis should not be based on hyperammonemia alone; it requires integration of the neurological syndrome, competing causes, drug exposure, and response to treatment.

In this case, phenytoin toxicity, aspiration pneumonia with respiratory failure, sedative polypharmacy, and catabolic stress were meaningful competing contributors. Persistent encephalopathy and hyperammonemia after correction or improvement of these factors, followed by neurological and biochemical recovery during valproate taper and after withdrawal, supported an important causal contribution from valproate. Withdrawal required continued seizure surveillance and subsequent addition of topiramate after seizure recurrence. Careful temporal assessment can therefore support both recognition and safe management of this reversible complication in patients receiving complex antiseizure polytherapy.

## References

[1] Wadzinski J, Franks R, Roane D, Bayard M (2007). Valproate-associated hyperammonemic encephalopathy. J Am Board Fam Med.

[2] Chopra A, Kolla BP, Mansukhani MP, Netzel P, Frye MA (2012). Valproate-induced hyperammonemic encephalopathy: an update on risk factors, clinical correlates and management. Gen Hosp Psychiatry.

[3] Lin K, Cao VF, Au C, Dahri K (2022). Clinical pharmacokinetic monitoring of free valproic acid levels: a systematic review. Clin Pharmacokinet.

[4] Tseng YL, Huang CR, Lin CH (2014). Risk factors of hyperammonemia in patients with epilepsy under valproic acid therapy. Medicine (Baltimore).

[5] Vaidyanathan S, Chatorikar S, Praharaj SK, Munoli RN, Udupa ST (2023). Valproate-associated hyperammonemic encephalopathy: clinical correlates and management strategies in a tertiary care center. J Clin Psychopharmacol.

[6] Wong YJ, Fan J, Wan A, Mihic T, Gnyra M (2023). Valproic acid-associated hyperammonemia: a systematic review. J Clin Psychopharmacol.

[7] Gziut T, Thanacoody R (2025). L-carnitine for valproic acid-induced toxicity. Br J Clin Pharmacol.

[8] Häberle J, Burlina A, Chakrapani A (2019). Suggested guidelines for the diagnosis and management of urea cycle disorders: first revision. J Inherit Metab Dis.

[9] Baddour E, Tewksbury A, Stauner N (2018). Valproic acid-induced hyperammonemia: Incidence, clinical significance, and treatment management. Ment Health Clin.

[10] McMorris T, Chu A, Vu L, Bernardini A (2021). Hyperammonemia in patients receiving valproic acid in the hospital setting: a retrospective review. Ment Health Clin.

